# The Evolution of Sex-Related Traits and Genes

**DOI:** 10.4061/2011/807218

**Published:** 2011-09-12

**Authors:** Alberto Civetta, José M. Eirín-López, Rob Kulathinal, Jeremy L. Marshall

**Affiliations:** ^1^Department of Biology, University of Winnipeg, Winnipeg, MB, Canada R3B 2E9; ^2^Department of Cell and Molecular Biology, University of A Coruña, E15071 Coruña, Spain; ^3^Department of Biology, Temple University, Philadelphia, PA 19122, USA; ^4^Department of Entomology, Kansas State University, Manhattan, KS 66506, USA

A growing number of studies are drawing attention to a consistent pattern of rapid evolution of sex-related traits and genes. Different hypotheses have been proposed to explain this rapid divergence. For traits and genes directly involved in reproduction, the pressures imposed by different sexes are likely to have driven rapid evolution, either by conflictive interactions, forms of female cryptic choice (female sex drive), or male differential use of traits and behavioral tactics (male sex drive). However, other factors such as subfunctionalization linked to gene duplications, birth and death processes, and pleiotropic effects, particularly those linked to immunity, can also drive fast change. The disruption of reproductive traits and gene interactions are of interest to those studying speciation because they can impede gene flow among diverging populations. Different studies have also shown that rapid changes and adaptive diversification between species can occur in reproductive developmental processes such as sex determination and gamete formation. Finally, high-throughput molecular technologies and bioinformatics approaches are allowing us to place patterns of gene evolution within the broad context of whole genome dynamics.

The ten papers published in this Issue tackle some of these fundamental questions on the evolution of sex-related traits and genes. Three opening review articles address problems relating to testing sexual selection at the molecular level, gene duplications and the evolution of sex bias patterns of gene expression, and the evolutionary origin of gametogenesis. A. Wong reviews recent studies in rodents and primates that have tested for associations between proxies of sexual selection and rates of evolution on male reproductive genes. He highlights how only a few studies have been able to establish significant associations. While this might cast some doubts on the role of sexual selection on the rapid evolution of male reproductive genes, he brings up some of the analytical problems linked to these prior studies and the need to incorporate multigene analysis and population genetics-based approaches. In their review, M. Gallach and collaborators address the problem faced by sexually reproducing species due to the need to generate two sexes with different patterns of tissue-specific gene expression and physiologies. They show that this conflict between sexes has led to a nonrandom distribution of sex-biased genes in different species and that gene duplication can help avoid sex-related genomic clashes leading to dosage compensation or meiotic sex chromosome inactivation as well as alleviate sexual conflict within species. Lastly, J. M. Eirín-López and J. Ausió provide an up-to-date insight into the evolutionary origins of the molecular mechanisms underlying sexual reproduction in metazoan animals. By bringing attention into the recent characterization of the DAZ family of reproductive proteins, the authors suggest that the wide conservation of a core reproductive machinery encoded by premeiotically expressed genes across Metazoa lends support to a common evolutionary origin for gametogenesis, rejecting the hypothesis that gametogenesis evolved multiple times in different lineages.

 Seven research articles also address a variety of questions related to the evolution of sex-related traits and genes. Fox et al. reanalyzed data from seven human populations to show preferential paternal grandmothering behavior towards granddaughters. They conclude that such behavior is likely driven by selection at selfish X-linked gene/s for alleles that increase the survival of grandmothers, because grandmothers who live longer provide longer care and benefits for their granddaughters. Thus, this work demonstrates that grandmaternal care accounts for the evolution of increased longevity in humans and is influenced by selection on the X-chromosome rather than by paternity uncertainty. Nelson and Neiman compared copulatory behavior in young and old asexual lineages of a freshwater snail. Contrary to an expected vestigialization of costly mating behaviors, the author found no differences in female copulation frequencies based on lineage age. Their results provide a rather cautionary warning regarding broad assumptions about the evolution of sex-related traits that are commonly linked to very complex aspects of the organism's biology. Two papers in this Issue deal with postcopulatory traits and genes. Greenspan and Clark tested differences in sperm competitive ability among strains of *Drosophila melanogaster* and found significant associations with polymorphisms residing on X-chromosome candidate genes. While these studies have traditionally been conducted by focusing on autosomal genes for seminal fluid proteins (SFP), their results suggests that such an approach has been biased and that we have been missing a large proportion of male reproductive variation linked to genes other than SFP. Sagga and Civetta surveyed divergence between species of Drosophila on noncompetitive postmating reproductive phenotypes and found evidence for a strong postmating prezygotic isolation (PPI) barrier among species of the virilis subgroup. They further established that PPI is due to reduced fertilization rates despite normal rates of sperm transfer and egg-laying. An inspection of sperm within female storage organs revealed low retention of heterospecific sperm leaving open the question of how female and female-male interactions might contribute to the exclusion of heterospecific sperm from storage. The paper by Jagadeeshan et al. also deals with the topic of species isolation and speciation in Drosophila. The authors analyzed 4,843 genes in 5 *D. melanogaster* subgroup species, seeking loci that exhibit accelerated rates of evolution associated with speciation. The finding that the majority of sex and reproduction-related genes are persistently subject to rapid evolution leads the authors to propose that selection, in combination with rapid demographic changes, makes a critical contribution to the elevated rates of evolution during speciation. 

Finally, two papers look at larger macroevolutionary patterns of sex-bias evolution. Grassa and Kulathinal provide a functional and comparative genomic approach to understanding the role of sex-related genes in vertebrates. Using available ESTs from a range of tissues, fully assembled genomes, and well-curated gene models from five diverse vertebrate species, the authors characterize evolutionary rates in close to 5,000 reproductive and non-reproductive orthologs. Gonadal-specific genes that are lineage-specific show the greatest rate of evolutionary change. Surprisingly, an opposite trend emerges on functionally conserved orthologs. Kimball et al. catalog the presence and absence of sexually dimorphic characters involved in sexual signaling across the avian taxonomic clade, Phasianidae, which include pheasants and partridges. Six out of the nine dimorphic characters are visual cues while the remaining three relate to competitive ability. A variety of intersexual theories are described to explain the gains and losses of these sexually dimorphic characters in this lineage. 

The evolution of sex-related traits and genes has clearly become a diverse and dynamic area of research and we hope that the articles included in this Issue will inspire, motivate, and challenge others to study the many questions that are emerging in this boundless field.

